# TEM, SEM, and STEM-based immuno-CLEM workflows offer complementary advantages

**DOI:** 10.1038/s41598-020-79637-9

**Published:** 2021-01-13

**Authors:** Viola Oorschot, Benjamin W. Lindsey, Jan Kaslin, Georg Ramm

**Affiliations:** 1 Ramaciotti Centre for Cryo EM, Monash University, Melbourne, VIC 3800 Australia; 2 Australian Regenerative Medicine Institute, Monash University, Melbourne, VIC 3800 Australia; 3 Department of Biochemistry, Biomedicine Discovery Institute, Monash University, Melbourne, VIC 3800 Australia; 4 Present Address: European Molecular Biology Laboratory, Electron Microscopy Core Facility, Heidelberg, Germany; 5 Present Address: Department of Human Anatomy and Cell Science, Rady Faculty of Health Sciences, University of Manitoba, Winnipeg, R3E 0J9 Canada

**Keywords:** Imaging, Microscopy, Biological techniques, Cell biology, Neuroscience, Stem cells, Anatomy

## Abstract

Identifying endogenous tissue stem cells remains a key challenge in developmental and regenerative biology. To distinguish and molecularly characterise stem cell populations in large heterogeneous tissues, the combination of cytochemical cell markers with ultrastructural morphology is highly beneficial. Here, we realise this through workflows of multi-resolution *immuno*-correlative light and electron microscopy (*i*CLEM) methodologies. Taking advantage of the antigenicity preservation of the Tokuyasu technique, we have established robust protocols and workflows and provide a side-by-side comparison of *i*CLEM used in combination with scanning EM (SEM), scanning TEM (STEM), or transmission EM (TEM). Evaluation of the applications and advantages of each method highlights their practicality for the identification, quantification, and characterization of heterogeneous cell populations in small organisms, organs, or tissues in healthy and diseased states. The *i*CLEM techniques are broadly applicable and can use either genetically encoded or cytochemical markers on plant, animal and human tissues. We demonstrate how these protocols are particularly suited for investigating neural stem and progenitor cell populations of the vertebrate nervous system.

## Introduction

In recent years, correlative light and electron microscopy (CLEM) has bridged the use of fluorescent proteins and immunolabelling with high-resolution EM^1,2^. This methodology has permitted description of the cellular detail of rare structures and events^3–6^ and given rise to multiple protocols for widespread use in biology^7–9^. However, with few exceptions^10–12^, most protocols using CLEM do so using plastic resins such as Lowicryl or Epon, that considerably limit antibody penetration and staining on tissue sections^2^. We and others have previously demonstrated that performing *immuno*-CLEM (hereafter referred to as *i*CLEM) using Tokuyasu cryo-preparation of tissue samples significantly enhances fluorescent labelling of probes to provide high-resolution TEM of specific tissue regions of interest^4,13^. *i*CLEM is also suitable to identify specific regions of interest within small organisms^4^. To apply *i*CLEM to identify, visualize and quantitate larger tissue regions we set out to establish an EM pipeline that allows to progressively work from fluorescence microscopy to low and high EM magnifications. As a model system for this workflow, we use the identification of tissue stem cells in zebrafish brain.

Tissue stem cells play an important function in organ development, maintenance and growth. Their role as a potential source of endogenous repair, in particular during adulthood, has further been established over the last decade^14–16^. A fundamental goal of stem cell biology is to identify candidate stem and progenitor populations that contribute and respond to the needs of specific organ systems under physiological and pathological states. However, within a single tissue of interest, it remains a major challenge to identify and characterise closely related cells as they progress through distinct lineages and how these lineages are modified with disease or regeneration^17–20^. Global observations of changes in large tissue domains at high resolution, such as the re-organization of the stem cell niche, is even further out of reach. Neither classical transmission electron microscopy (TEM), nor antibody labelling alone, have been sufficient to date to describe the diversity of stem and progenitor phenotypes that construct, remodel, and regenerate organ tissues. Being able to correlate diverse cell states and morphologies to cells identified by expression of specific markers is therefore fundamental to further our understanding of cell plasticity in tissues.

In this paper, we present new *i*CLEM Scanning EM (SEM) and *i*CLEM Scanning Transmission EM (STEM) protocols that take advantage of the antigenicity afforded by Tokuyasu sample preparation^21^. We further extend these techniques to our previously developed *i*CLEM-TEM methodology, showcasing the ability to combine *i*CLEM-TEM with subsequent immuno-gold labelling of multiple proteins of interest in consecutive sections. To demonstrate the application of our *i*CLEM pipeline, we examine tissue from the adult zebrafish telencephalon following a forebrain lesion^22^—a neural stem cell niche recognized for its diversity of cellular profiles^23–25^ and regenerative potential of local neural stem and progenitor cells (NSPCs)^22,24,26,27^. The small size of the adult zebrafish brain additionally allows the entire forebrain to be embedded in gelatine blocks and the complete dorsal telencephalic niche to be visualized in sections, making this model highly amenable to our *i*CLEM approach to identify new subclasses of NSPCs.

Our work provides a side-by-side comparison of *i*CLEM using Tokuyasu cryo-embedding across three different microscopy methods (SEM, STEM, TEM; see Fig. 8) to distinguish cell populations at increasingly higher cellular resolution. Specifically, our results highlight multiple advantages of this *i*CLEM workflow: (1) *i*CLEM-SEM on 200 nm semithin sections provides a quantifiable overview of large tissue regions that does not suffer from the presence of grid bars and benefits from the use of thicker sections to obtain strong immunofluorescent (IF) labeling; (2) *i*CLEM-STEM on ultrathin sections (i.e. 70 nm) has the advantage of getting a higher resolution overview of the tissue section compared to *i*CLEM-SEM; (3) stitching multiple images using montage and correlative software for SEM and STEM is a useful tool for correlation and navigation in large tissue samples; and (4) *i*CLEM-TEM provides high resolution ultrastructural details of cells with the ability to examine consecutive tissue sections with different labelling techniques. We expect the *i*CLEM pipeline presented here to be of broad interest and practical use for distinguishing cell types or states in plant and animal models alike, and an essential methodology to define NSPC-specific behaviour in the adult zebrafish model in health and disease.

## Results

### Immuno-CLEM can be used to examine large sections of adult zebrafish brain

In this study, we aimed to establish and compare different *i*CLEM workflows and apply them to the characterisation of unique NSPC populations. We used tissue from the adult forebrain telencephalon, where the pallial stem cell niche borders the telencephalic ventricle dorsolaterally^23,25^. The telencephalic neurogenic niche of the zebrafish, in particular, has been the focus of intense study in the healthy and injured brain, owing to its heterogeneous NSPC populations and remarkable neural regenerative potential^22–32^. Here, we focus on the identification and morphological characterisation of three cell types within the telencephalon. Non-cycling radial-glia-cells are categorised by the expression of Glutamine Synthase (GS) and proliferative cells by Proliferating Cell Nuclear Antigen (PCNA) driven GFP-expression, while proliferating radial-glia cells have both markers.

**Figure 1:**
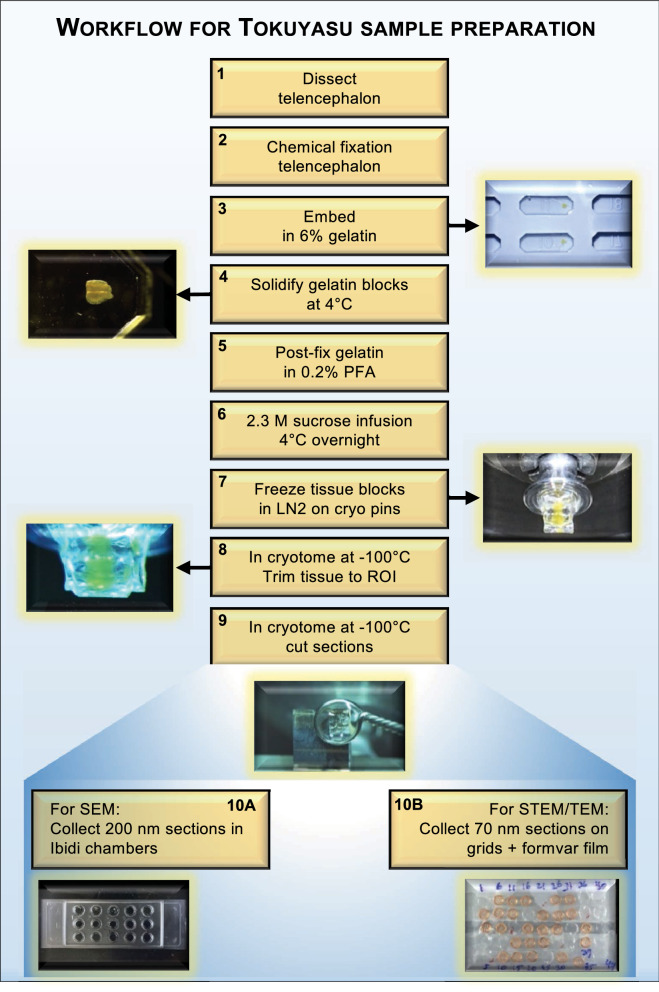
Overview of workflow (*steps 1–10*) for Tokuyasu sample preparation for subsequent SEM or STEM/TEM tissue processing. Images highlight examples of key steps in workflow using the adult zebrafish forebrain. Note that step 10A is the endpoint for transitioning to *i*CLEM-SEM processing, while 10B is the transition point for subsequent *i*CLEM-STEM/*i*CLEM-TEM processing. PFA, paraformaldehyde; ROI, region of interest.

Unlike the larger brains of rodents, the small size of the adult zebrafish CNS permits the entire intact forebrain to be chemically fixed and embedded in gelatin blocks for the Tokuyasu method (Fig. 1). Moreover, penetration of fixatives into forebrain tissue is facilitated by the small size of samples. Notably, fixation, embedding, and cutting of Tokuyasu cryo-ultramicrotome sections follows the same procedure for all workflows used below. Subsequent cryo-sections (Fig. 1—step 9) can be collected on either the surface of Ibidi chambers (SEM; Fig. 1—step 10A) or directly on EM grids coated with formvar film (STEM/TEM; Fig. 1—step 10B) to allow both left and right hemispheres to be captured for a continuous workflow from low to high magnification. This approach permits comparisons between hemispheres using *i*CLEM-SEM (large multi-cell tissue overviews of a region of interest), *i*CLEM-STEM (combination of tissue overview with greater morphological detail), *i*CLEM-TEM (ultrastructure detail of a cell type of interest) workflows (see overview of applications in Fig. 8), and to uncover how the intact stem cell niche is constructed over development and remodelled after injury.

### iCLEM using SEM offers a method to visualize large tissue areas for qualitative or quantitative analysis

Adult neurogenic niches of vertebrates, including the zebrafish, are extensive and cover a large surface area composed of heterogeneous NSPCs. Having a method to visualize the association between distinct populations along with their morphological detail would greatly facilitate the understanding of the overall niche architecture and how the niche is remodelled following perturbation. Our earlier work has shown that based on ultrastructural features, upwards of 6 distinct morphologies of cells compose most zebrafish stem cell niches^25,33^, though it is possible more subtypes exist. In the zebrafish brain, two major classes of NSPCs are present in different combinations across adult stem cell niches, including radial-glial cells and neuro-epithelial cells^31^. The large majority of radial-glial cells reside in a non-proliferative (i.e. quiescent) state, but in some niches neighbour a small population of cycling radial-glia. Specifically within the dorsal forebrain, viewed in cross-section radial-glial cells reside along the telencephalic ventricle. This cell population is commonly identified by the intermediate filament marker Glial Fibrillary Acidic Protein (GFAP) or the enzyme Glutamine Synthetase (GS) where expression is seen both in the cytoplasm and elongated processes of radial-glial cells. By combining the above glial markers with Proliferating Cell Nuclear Antigen (PCNA) that labels the G_1_ to M phase of the cell cycle^34,35^, it is further possible to discriminate actively dividing radial-glial from those that are quiescent. At present, the complete lineage of radial-glial cells still remains unclear. In the midbrain, recent evidence has shown that at least three cell types make up the neuro-epithelial lineage, including slow and fast amplifying populations^36^. Whether this same lineage holds true in other brain regions where these NSPCs reside remains to be explored. Important, both cell types play a central role in both constitutive and regenerative neurogenesis^32^.

**Figure 2:**
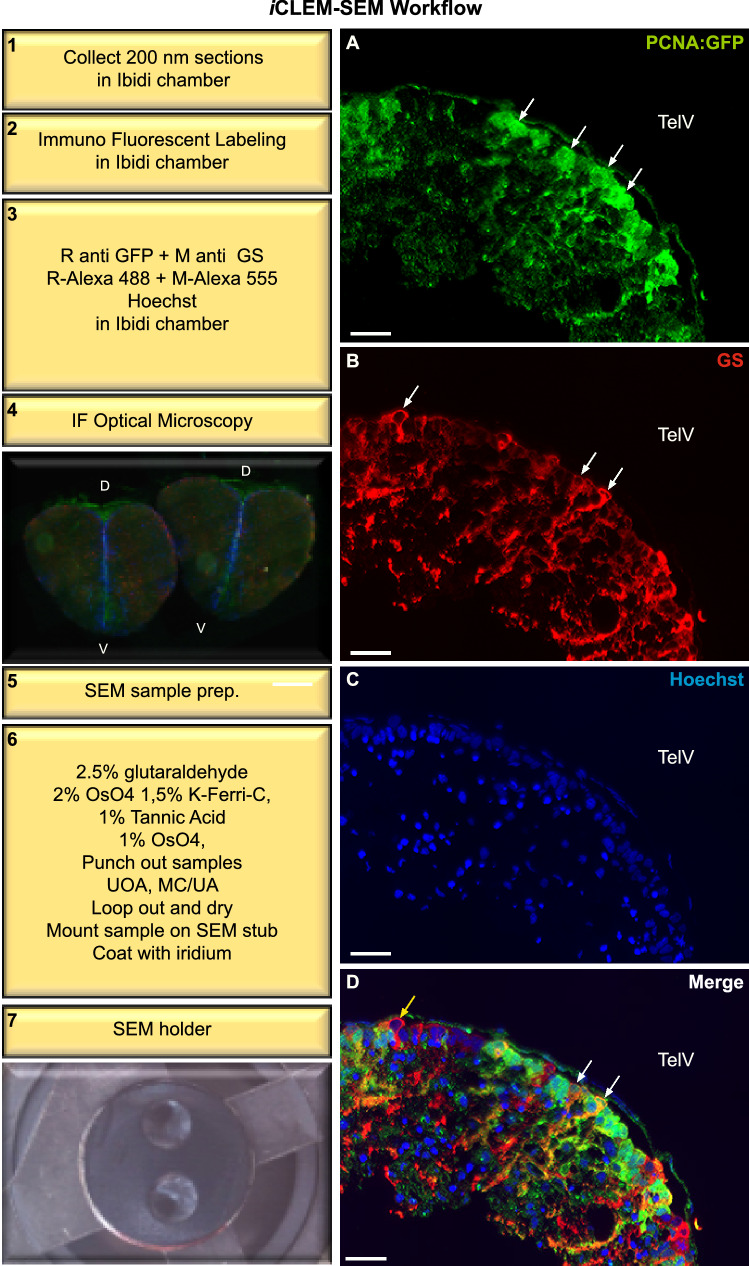
*i*CLEM-SEM workflow and immunofluorescent labelling on Tokuyasu chemical-fixed cryosections. Left: Major steps required for *i*CLEM-SEM workflow (steps 1–6). (**A**) Green Fluorescent Protein (GFP)-positive labelling (*green*) of proliferating cells from the Tg(PCNA:GFP) transgenic line (white arrows). (**B**) Glutamine Synthetase (GS)-positive labelling (*red*) of glial cells (white arrows). (**C**) Hoechst counterstaining (*blue*). (**D**) Multi-channel merge showing co-labelling of PCNA:GFP-positive cells with the glial marker (*orange cells; white arrows*) in the cytoplasm of a small number of cells. Differences in the intensity of antibody staining can additionally be noted (compare white arrows and yellow arrow). *D* dorsal; *V* ventral. *TelV* telencephalic ventricle. Scale bar (**A**–**D**) 20 µm.

A general drawback of EM is that the tissue area that can be investigated is very small. In addition, larger regions of interest are often at least partially concealed by the grid bars of the EM grid. To overcome these limitations, we developed an *i*CLEM method to capture large tissue overviews. The method combines fluorescent light microscopy with scanning electron microscopy (SEM) and makes use of standard SEM holders. The entire adult zebrafish brain is smaller than the size of a diamond knife used for cryo-sectioning. This allows cutting the whole brain and interrogation of the entire stem cell niche for analysis. The technique involves the use of Ibidi chambers as a substrate for both immunofluorescence (IF) and SEM (Fig. 2—step 1). The ability to immuno-label and analyze multiple tissue sections within wells of a single Ibidi chamber using this *i*CLEM-SEM approach greatly expands options for tissue analysis and quantification^37,38^.

Our results demonstrate that correlative immuno-labelling for antibodies against Green Fluorescent Protein (GFP) labelling proliferating cells and the radial-glial marker, GS, can be successfully completed and imaged on 200 nm thick sections in Ibidi chambers (Fig. 2—step 2–3). Owing to the use of the Tokuyasu method, excellent IF signal of conjugated secondary antibodies can be obtained across large tissue regions of the adult pallial stem cell niche. Immuno-labelling reliably detects GFP from the Tg(PCNA:GFP) line in the cell nucleus as well as the cytosol (Fig. 2A; white arrows). Moreover, the cytoplasmic nature of GS-labelling along with radiating processes of radial-glial cells towards the central parenchyma of the pallium can be clearly observed (Fig. 2B; white arrows). This illustrates that the normal pattern of GS^+^ and PCNA^+^ immunolabeling of proliferative and non-proliferative radial-glia observed in cryosectioned tissue is similarly detected with high sensitivity and fidelity using Tokuyasu sample preparation. In addition, general tissue architecture and anatomical landmarks can be visualised using common nuclear markers, such as Hoechst (Fig. 2C). Importantly, individual markers can be easily overlaid to study co-labelling of NSPC populations of interest (Fig. 2D). The staining pattern observed in Fig. 2D using IF labelling in Tokuyasu prepared tissue is highly consistent with the use of this same complement of antibodies with standard immunohistochemistry in cryosectioned tissue of the dorsal telencephalon^22,23^. Furthermore, the sensitivity of IF labelling on Tokuyasu prepared sections can detect even subtle differences in non-proliferating (GS^+^/PCNA:GFP^-^; Fig. 2D, yellow arrow) and proliferating (GS^+^/PCNA:GFP^+^; Fig. 2D, white arrows) glial cells, as evidenced by different intensities of Alexa-555 labelling of resident radial-glia at the telencephalic ventricular zone. It should be noted that due to the use of cytoplasmic markers on thin sections used for confocal microscopy (thickness below resolution of conventional light microscopy), the immunofluorescence pattern appears different from standard specimens.

An advantage of our *i*CLEM-SEM technique is that multiple serial sections can be labelled for IF and subsequently processed for SEM within a single tissue region of interest (Fig. 2—step 4–5). As the same tissue cells are present in many consecutive ultrathin sections, cell characteristics can potentially be tested with a large number of different IF antibodies (i.e. different antibodies/section) and correlated to EM ultrastructure. To stabilize the structural integrity of the sections and obtain high contrast required for SEM, we postfixed and stained the sections by successive steps in glutaraldehyde, reduced osmium, tannic acid, and additionally osmium tetroxide. To allow efficient embedding and drying of the section, the area containing the sections was punched out of the Ibidi chamber, stained with uranylacetate and dried in a mixture of methylcellulose and uranylacetate. Tissue samples were then mounted on a standard SEM stub, coated with iridium or platinum, and placed on an SEM holder (Fig. 2—steps 5–7). This method results in good ultrastructural contrast for backscattered electron imaging of the tissue sections at both low resolution to obtain overviews as well as for detailed high resolution imaging. *i*CLEM using SEM does not require the need to mount tissue sections on grids, and provides a larger viewing area without obstruction by grid bars.

**Figure 3:**
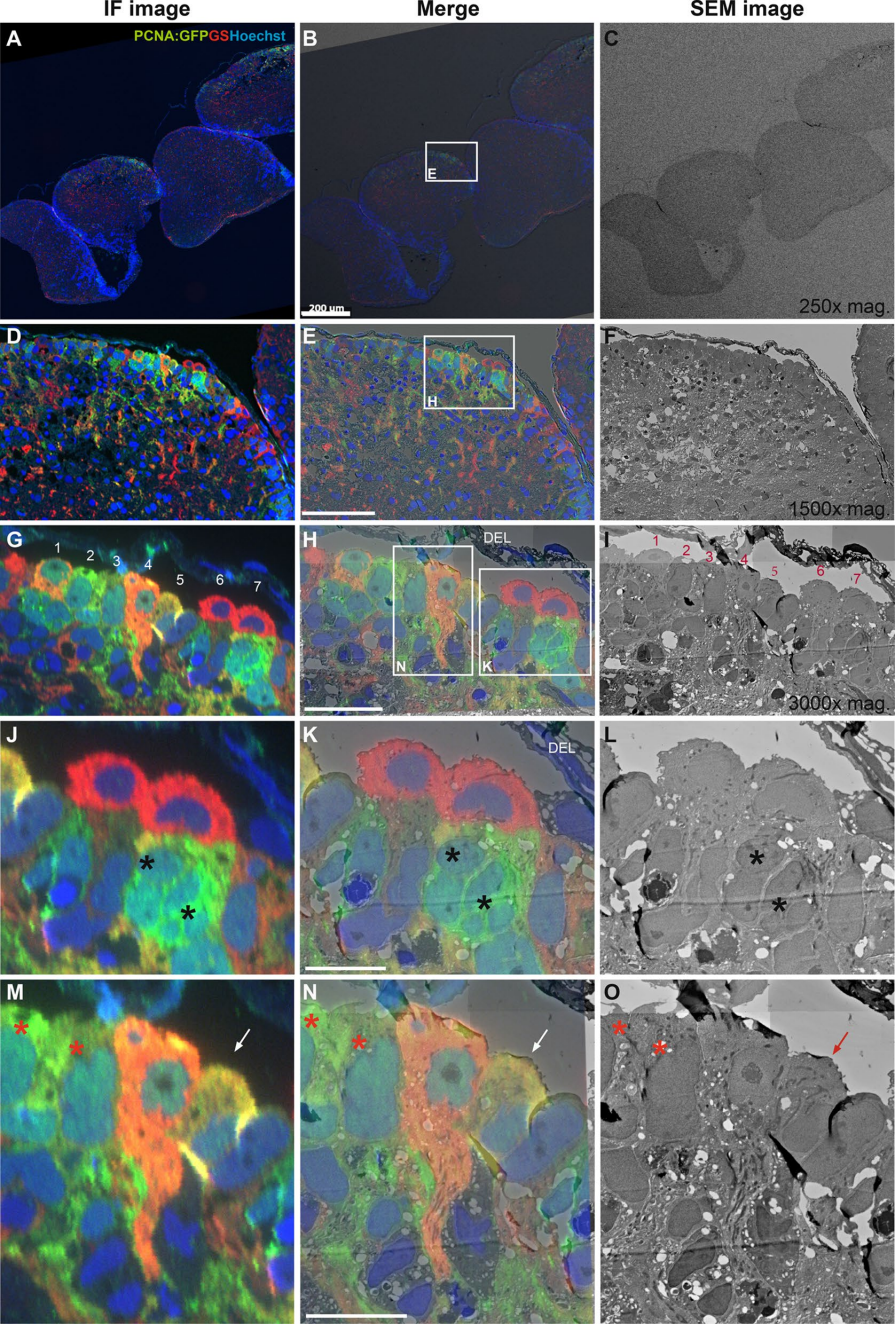
*i*CLEM imaging using SEM. Merged images (middle column) illustrate exact overlay of immunofluorescent (IF) images (left column) and SEM images (right column) using MAPS software. (**A–C**) Low 250X magnification images of adult zebrafish forebrain cross-section illustrating ability to capture multiple whole brain sections. White box in (**B**) depicts region displayed in (**D–F**). (**D–F**) Mid-dorsal view of zebrafish pallium showing dense row of distinctly labelled cells below the dorsal ependymal lining (DEL—see panel H). White box in (**E**) depicts region displayed in (**G–I**). (**G–I**) Basic morphological details of individual cells (1–7) positioned at the telencephalic ventricle using both IF and EM resolution. White boxes in (**H**) depict cell types shown at higher magnification in subsequent panels (**J–L**) and (**M–O**). (**J–L**) Example of nuclear staining in GS^+^ radial-glial cells (*red*) and putative proliferating GFP:PCNA^+^ progenitor cells (*green*; black asterisk). (**M–O**) Example of a PCNA:GFP^+^/GS^+^ proliferating radial-glial cell (*orange*) showing an extending glial process compared with neighbouring cycling cells (*green*; red asterisk) and cell in a potential transitional state (*yellow*; white/red arrow). Note slightly out-of-focus images in panels (**J–O**) indicating limit of resolution using SEM. Scale bar (**B**) 200 µm; (**E**) 70 µm; (**H**) 20 µm; (**K**,**N**) 10 µm.

Using correlative software packages such as the MAPS software on a FE-SEM (Nova Nano SEM 450), our data show that regions of interest can easily be selected from multiple *i*CLEM-SEM processed tissue sections (Fig. 3A–C) for visualization and analysis at higher SEM magnification (Fig. 3D–F). At magnifications of approximately 1500X, quantifications can be performed based on IF details, SEM morphology or a combination of both using merged images of tissue subregions (Fig. 3G–I). At a magnification of 3000X significant ultrastructural detail of cytoplasmic organelles can additionally be extracted for each immuno-labelled cell type using the *i*CLEM-SEM method (Fig. 3J–O).

Our examination of NSPCs positioned adjacent the telencephalic ventricle show at least three different cellular morphologies, including proliferating radial-glia (yellow to orange GS^+^/PCNA:GFP^+^; cell #1, 4, 5), proliferating non-glia (green GS^-^/PCNA:GFP^+^; cell #2–3), and non-cycling radial-glia (red GS^+^/PCNA:GFP^-^; cell #6–7) cell populations (Fig. 3G–I). Interestingly, the lower GS^+^ expression observed in the single GS^+^/PCNA:GFP^+^ cell that appears yellow (see Fig. 3M–O, white/red arrow) suggests that it could be a transitional state between non-cycling GS^+^/PCNA:GFP^-^ cells (red) and proliferative radial-glia (orange). In agreement, the nucleus and cytoplasm of the yellow radial-glia (Fig. 3M, white arrow) is much more closely related to non-proliferative GS^+^/PCNA:GFP^-^ cells (red cells; Fig. 3J–L) compared with that of mitochondrial-rich cycling radial-glia (orange) that contain a prominent nucleolus (orange cell; Fig. 3M–O). Importantly, our *i*CLEM-SEM results illustrate that the cell size of putative GS^-^/PCNA:GFP^+^ progenitor cells located in the subventricular zone (Fig. 3J–L; black asterisk) are considerably smaller than those GS^-^/PCNA:GFP^+^ cell populations positioned at the ventricle (Fig. 3M–O; red asterisk). These potential progenitors contain less surrounding cytoplasm and irregular nuclei, and appear to be found in clusters similar to migrating neuroblasts (type A cells) in the mammalian subventricular zone^39–41^. This cellular profile is noticeable when contrasted against the larger, ventricular localized PCNA^+^ cells that have larger and more ovoid nuclei (Fig. 3M–O; red asterisk).

### iCLEM using STEM offers a method to prepare correlative overviews and high resolution images of tissue

**Figure 4:**
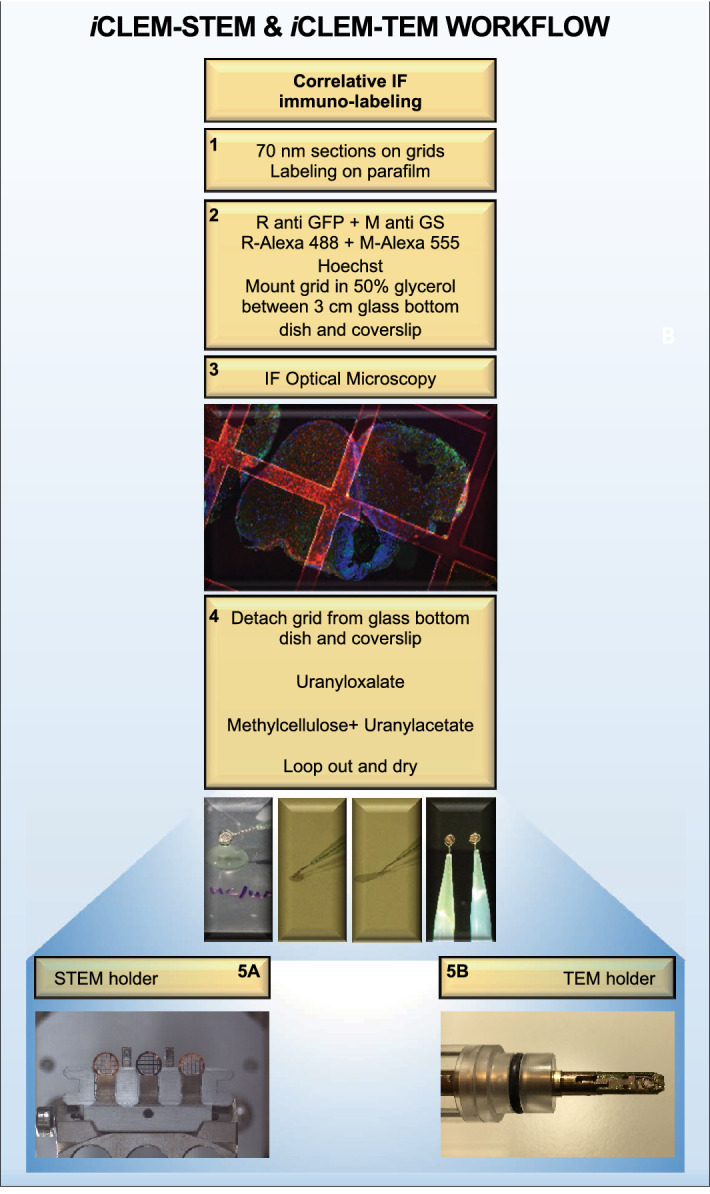
*i*CLEM-STEM and *i*CLEM-TEM workflow. Workflow of major tissue processing steps (1–5) common to *i*CLEM-STEM and *i*CLEM-TEM following Tokuyasu sample preparation. Once grids are looped out and dried for EM imaging, grids are placed on either a STEM holder (step—5A) or TEM holder (step—5B). Note that for STEM imaging, a specialized STEM holder for grids compatible with SEM in addition to a bottom detector positioned below the sample is required. Importantly, STEM samples are congruent with TEM samples and can be imaged sequentially if it is desirable to visualize the same sample at both resolutions. *IF* immunofluorescence.

Although *i*CLEM-SEM has the advantage of providing large tissue overviews, at magnifications above 6000X it begins to suffer from reduced imaging quality. However, this can be circumvented by applying *i*CLEM-STEM and *i*CLEM-TEM that both allow for substantially higher resolution imaging. The workflow for sample preparation for *i*CLEM-STEM and *i*CLEM-TEM are identical, and need to be adapted from *i*CLEM-SEM. Only thinner sections are suitable and the substrate has to be electron transparent (Fig. 4). STEM imaging is known to give TEM like images and resolution but with the option of providing large overviews of the tissue^42,43^. By using the correlative functions of the MAPS software on a Nova NanoSEM to obtain large overviews, we explored the STEM capabilities of the SEM. Using ultrathin 70 nm sections for STEM provides substantially better resolution at magnifications above 3000X compared to the thick 200 nm sections used for SEM.

**Figure 5:**
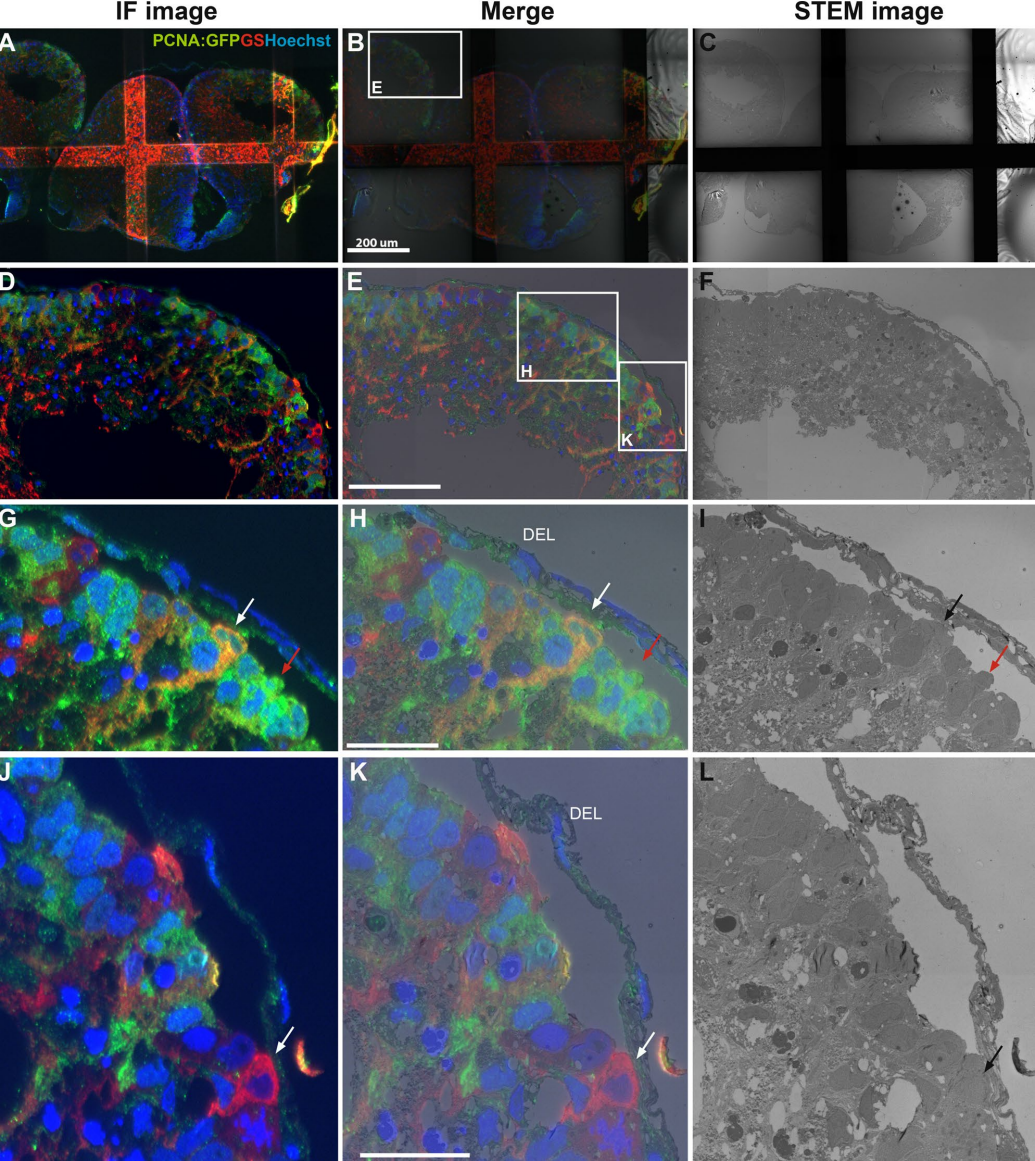
*i*CLEM imaging using STEM. Merged images (middle column) illustrate exact overlay of immunofluorescent (IF) images (left column) and STEM images (right column) using MAPS software. (**A–C**) Reconstructed tile-sets of STEM images of the adult zebrafish forebrain in cross-section on grids bars using MAPS software at low resolution. White box in (**B**) depicts region displayed in (**D-F**). (**D-F**) Dorsal view of zebrafish pallium showing dense row of labelled cells below thin dorsal ependymal lining (DEL—see panel H). White boxes in (**E**) depict regions displayed at higher magnification in (**G–I**) and (**J–L**). (**G–I**) GS^+^/PCNA:GFP^+^ radial-glial cell (*orange*; white/black arrow) contrasted with a neighbouring GS^-^/PCNA:GFP^+^ cycling cell (*green*; red arrow). (**J–L**) Example of a GS^+^/PCNA:GFP^−^ cell profile (*red*; white arrow). Scale bar (**B**) 200 µm; (**E**) 80 µm; (**H**,**K**) 20 µm.

Our results demonstrate that the use of thin 70–80 nm Tokuyasu cryo-sections on grids immuno-labelled with two antibodies and corresponding fluorescent markers, allows acquisition of medium resolution tile-sets (i.e. montage of multiple images) of complete tissue hemispheres in STEM mode (Fig. 5A–C). From these medium-resolution tile-sets, in conjunction with software such as MAPS, *i*CLEM-STEM provides the ability to zoom into the area of interest, while still being able to make a correlation with the fluorescent data (Fig. 5D–L). For instance, using the zoom function on tile-sets allows a co-labelled cycling radial-glial cell (orange GS^+^/PCNA:GFP^+^; white/black arrow) to be easily distinguished from a neighbouring cycling cell (green GS^-^/PCNA:GFP^+^; red arrow; Fig. 5G–I). By moving to other locations of the montage, different cell identities can be examined, such as non-cycling radial-glia cells (red GS^+^/PCNA:GFP^-^; white/black arrow; Fig. 5J–L). *i*CLEM-SEM permits the option of quantification, albeit with lower morphological detail. By contrast, *i*CLEM-STEM at medium magnification provides greater cellular information of a large tissue region (Fig. 5G–L) and functions as an excellent navigation tool when grids are transferred from STEM to TEM to obtain high resolution ultrastructural cellular detail.

### iCLEM using TEM offers a method providing high resolution images of individual cells and the ability to collect consecutive tissue sections of the same cell for different analyses

Transmission Electron Microscopy (TEM) continues to be the most commonly used EM method to investigate ultrastructural details of individual cells and cell organelles^13,44^. By combining TEM with *i*CLEM using the same workflow as for STEM (see Fig. 4), our data shows that IF imaging on grids can be used to identify the cell of interest for subsequent analysis of its cytoarchitectural features at high resolution at the EM level. Moreover, since *i*CLEM-TEM is performed on grids similar to *i*CLEM-STEM, individual cells and tissue regions can be correlated between the two methods to provide medium magnification overviews (*i*CLEM-STEM) with associated high magnification images of ultrastructural properties for classification of cell types under diverse experimental conditions.

**Figure 6:**
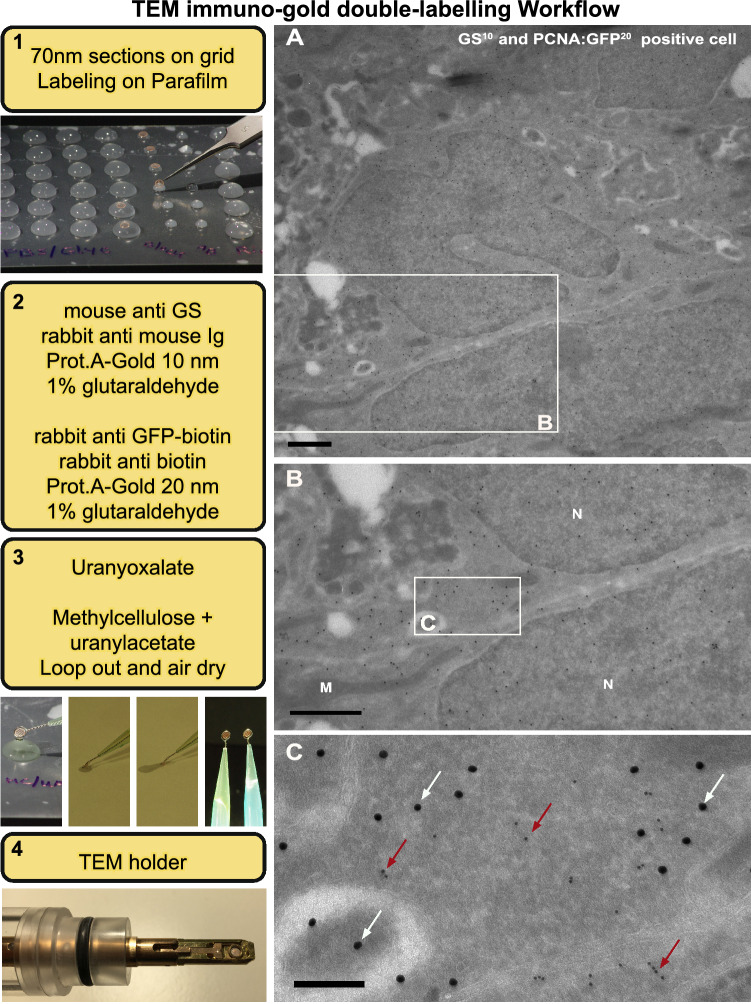
TEM immuno-gold double-labelling workflow and example of positive labelling on Tokuyasu chemical-fixed cryosections. Left: Major steps (1–4) required for TEM immuno-gold double-labelling workflow and associated images. (**A**,**B,C**) Cytosolic PCNA:GFP^+^/GS^+^ immuno-gold double-labelling at low (**A**) and high (**B,C**) magnifications demonstrating co-localization of the 10 nm (GS; red arrowheads) and 20 nm (PCNA:GFP; white arrowheads) particles. N, nucleus. Scale bar (**A**) 2 µm; (**B,C**) 200 nm.

As an extension of our *i*CLEM-TEM protocol, here we also introduce the use of consecutive sections at high resolution. Given the size of cells relative to the thickness of sections, cutting serial sections can be used to analyse a large number of cellular markers with both IF and immuno-gold in parallel. The additional use of gold-markers provides better correlation of cell-specific cellular markers in TEM. In particular, for the optimization of new protein markers of interest using *i*CLEM-TEM, having the ability to validate these markers in the same cell type using conventional immuno-gold labelling is of great value. Moreover, being able to analyse consecutive sections of the same cellular phenotype at high resolution further benefits studies of whole-cell morphometrics or investigation of cell organelles. Our established workflow for TEM immuno-gold double-labelling (Fig. 6, steps 1–4) illustrates that PCNA:GFP^+^ and GS^+^ labelling using two sizes of gold particles can be successfully completed and clearly discriminated in Tokuyasu prepared sections (Fig. 6A,B,C). This is demonstrated by the localization of 10 nm (GS, Fig. 6C red arrowheads) and 20 nm (PCNA:GFP, Fig. 6C white arrowheads) immuno-gold-particles in cell types of the adult zebrafish telencephalon known to be positive or negative for these proteins (Figs. S1 and S2; supplementary information).

**Figure 7:**
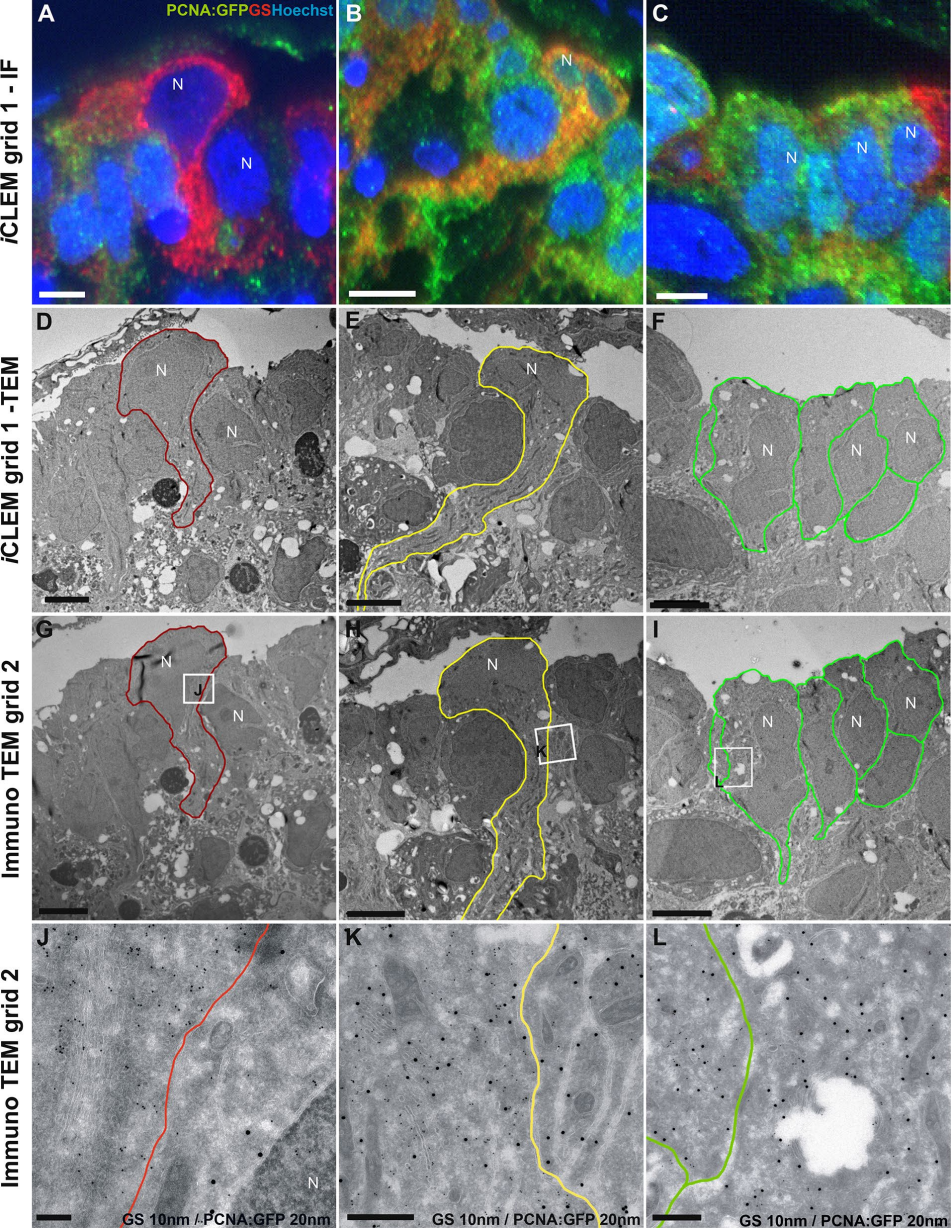
*i*CLEM imaging and consecutive immuno-gold labelling using TEM. The immunofluorescent (IF) and ultrastructural features of three morphologically distinct cell types (by column) in the adult zebrafish forebrain displayed using IF-labelling, TEM imaging, and immuno-gold TEM labelling. (**A–C**) IF-labelling demonstrates classification of cell types by fluorescent markers: non-proliferative radial-glia (**A**; PCNA:GFP^-^/GS^+^), proliferative radial-glia (**B**; PCNA:GFP^+^/GS^+^), and proliferating non-radial glia (**C;** PCNA:GFP^+^/GS^-^). (**D-F**) TEM imaging shows the unique morphological properties of each cell type on the same grid as shown in (**A–C**) (grid 1). Note the abundance of mitochondria in PCNA:GFP^+^/GS^+^ proliferative radial-glia (**E**). (**G–L**) Double-immuno-gold labelling of GS (10 nm) and GFP (20 nm) on a consecutive grid (grid 2) showing the specificity of IF markers that match seamlessly with antigen detection using more classical immuno-gold labelling. White boxes in G-I depict regions displayed at high magnification in (**J–L**). (**J–L**) High-magnification images showing distinct patterns of immuno-gold labelling consistent with fluorescent antibody labelling in (**A–C**). Note that serial sections from the same cell can be used to transition from IF to immuno-gold labelling. N, nucleus. Scale bars: (**A–I**) 5 µm; (**J**) 200 nm; (**K–L**) 500 nm.

Combining our *i*CLEM-TEM and TEM immuno-gold workflows provides a powerful method to conclusively identify tissue stem cells and characterize their ultrastructural features. Our results reveal that using our *i*CLEM-TEM method, consecutive 70 nm sections can be cut and placed on grids for immunofluorescent labelling (Fig. 7A–C; grid 1-IF) and subsequently imaged for TEM-level ultrastructural detail (Fig. 7D–F; grid 1-TEM). Moreover, adjacent 70 nm sections from the identical cell labelled for *i*CLEM-TEM can be processed by traditional double immuno-gold labelling (Fig. 7G–L; Immuno TEM grid 2). Accordingly, by cutting consecutive sections placed on sequential grids we are able to correlate two fluorescent markers with two (or up to three) Prot.A-Gold markers of different diameter (GS, 10 nm; PCNA:GFP, 20 nm) within the same cell (Fig. 7; compared A-C with G-L). The specificity of gold-labelling in corresponding regions of IF labelling (Fig. 7A–C) within cells can be seen in high magnification images in Fig. 7J–L. For example, non-cycling radial-glia with a GS^+^/PCNA:GFP^-^ immuno-positive profile (Fig. 7A—red cell) displays few PCNA:GFP^+^ 20 nm immuno-gold-particles as expected (Fig. 7J; left of red line). Conversely, double-immuno-labelling of proliferating radial-glia (Fig. 7B—orange cell) is reflected by a greater abundance of GS^+^ and PCNA:GFP^+^ immuno-gold-particles in this same cell type (Fig. 7K; left of yellow line). Collectively, combining IF labelling of antibodies with immuno-gold labelling using *i*CLEM-TEM provides unsurpassed information concerning the localisation of cell types relative to each other and the specificity of staining towards specific intracellular locations.

## Discussion

A number of *i*CLEM-TEM methods have been published in recent years with the aim of identifying hard-to-find organelles or rare events using correlative approaches^4,10,45–47^. However, there is no dedicated *i*CLEM method allowing examination of large tissue regions for the purpose of surveying complex cell populations as well as simultaneously identifying and examining ultrastructure of individual cells. We present a number of *i*CLEM methods to close this gap using different EM modalities including SEM, STEM, and TEM. We also take advantage of Tokuyasu tissue preparation for optimal labelling efficiency. Most existing *i*CLEM protocols have been developed using Lowicryl or EPON embedded tissue, which can be amenable to immunolabelling, but typically are challenging and produce much lower detectable immunofluorescence and immuno-EM labelling than what can be achieved using the Tokuyasu approach^2,11,48^. Tokuyasu sample preparation using chemical fixation was invented in 1973 by K. T. Tokuyasu and has been used across a wide range of plant and animal tissues^13,21,49^. The Tokuyasu approach retains high antigenicity by avoiding the use of denaturating solvents and embedding resins that prevent diffusion of antibodies and thus keeps proteins accessible for immuno detection^13^. The combination of Tokuyasu sections, immuno labelling, and CLEM is therefore a powerful approach for locating and targeting proteins or cells of interest.

To date Tokuyasu *i*CLEM has typically been used in conjunction with TEM^1,2,44^, although recently it has also been used for super-resolution optical microscopy in combination with SEM to label intracellular structures^37,50,51^. We start with using traditional SEM to image thick 200 nm semithin sections after prior immunofluorescence imaging. The protocol for Tokuyasu-SEM CLEM we present here is different from previous approaches^50,51^. Our protocol uses post-fixation performed after immunofluorescence to provide better preservation and integrity of large tissue areas leading to improvement in subsequent SEM visualisation. Our *i*CLEM-SEM workflow illustrates that the thicker 200 nm sections provide optimal fluorescence for CLEM. The sections can be readily stained and imaged within commercially available Ibidi chambers. A key advantage with this approach over tissue section collection on grids is the complete absence of grid bars, and therefore no data loss, as tissue sections on the Ibidi substrate are directly mounted onto a standard SEM holder. Furthermore, using Ibidi substrate maximises the area of tissue that can be visualised and is only limited by the size of the diamond knife. The *i*CLEM-SEM method presented here allows assessment of large tissue regions, but also provides the potential for 3-dimensional reconstructions. By scanning serially sectioned tissue, it is possible to reconstruct relative large tissue compartments for high resolution array tomography and 3-dimensional visualisation or analysis^52^. Both *i*CLEM-SEM and array tomography permit considerably greater tissue cross sections to be viewed compared with Focused Ion-Beam-SEM (FIB-SEM). FIB-SEM has a very small field of view and is used primarily for reconstructions of a single cell of interest^53,54^, rather than a more complex tissue domain such as a stem cell niche. Finally, it is important to highlight that both single tissue sections or serial sections produced using *i*CLEM-SEM can readily be exported to software programs such as Adobe Illustrator, FIJI/IMAGE J^56^, and CellProfiler, for cell tracing or other tissue-specific quantification methods. We envision that the practicality of *i*CLEM-SEM will lead to the development of new analytical approaches for researchers interested in investigating changes across large tissue regions during development, or between healthy and diseased states.

**Figure 8:**
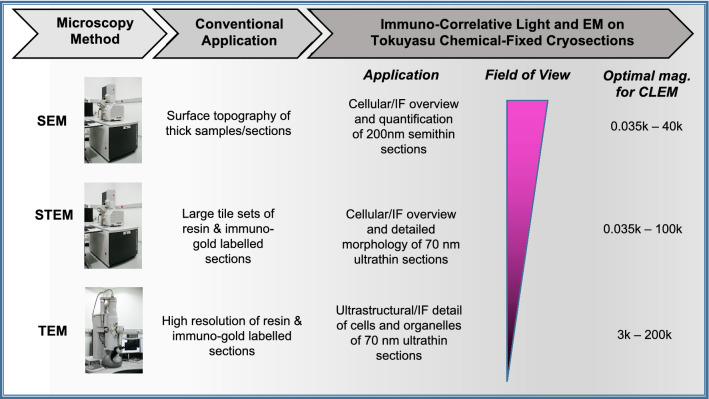
Conventional EM microscopy application compared with *i*CLEM on Tokuyasu prepared sections. Field of view depicts the slight overlap in magnification present between each microscopy method (SEM, STEM, TEM), and the overall decrease in field of view with the transitions from SEM-TEM resolution. The optimal magnification for our *i*CLEM technique for each microscopy method is shown in the right-most column, and may differ from magnification used for conventional EM imaging.

Our paper provides a method of combining *i*CLEM with EM data at a range of magnification that can be obtained on most current EM microscopes (Fig. 8). Our results comparatively demonstrate both the many benefits, but also the limitations, of these correlative approaches (Supplementary Table S1). While the *i*CLEM-SEM workflow does not allow for highest resolution imaging, the method can serve as a reference point for higher magnification *i*CLEM workflows using STEM or TEM. A notable advantage of using the STEM imaging mode is that it can also be performed on a SEM, making this method suitable for labs that only have access to a SEM, but not a TEM. We illustrate that by moving from SEM to STEM, higher resolution overviews can be obtained, with these two methods connected by the use of correlative SEM software. One caveat specific to *i*CLEM-STEM is that at high magnification imaging of Tokuyasu cryosections can clearly damage tissue due to the relative mild embedding used compared to EPON sections, which are more stable during STEM imaging. A drawback for both *i*CLEM-STEM and *i*CLEM-TEM is the presence of grid bars, which leads to the loss of information. However, it yields richer ultrastructural information derived at higher magnifications compared with SEM. Since STEM and TEM both rely on grids as their imaging substrate, the exact same grids can be used consecutively to study a tissue region, cell or organelle by STEM and TEM. The method allows different labelling combinations: (1) two antibodies with two corresponding fluorescent markers (Figs. 5, 7A–F), (2) two antibodies with two corresponding fluorescent markers and a single protein A-Gold marker, and (3) two antibodies with two corresponding protein A-Gold markers of different diameter (Figs. 6, 7G–L). Additionally, combining either method 1 or 2 with method 3 on consecutive grids permits correlation of fluorescent data with high resolution immuno-gold labelling. The different labelling methods at the correlative STEM/TEM levels and in combination with immuno-gold labelling allows precise interrogation of cellular features to conclusively identify unique cell phenotypes.

For correlation, here we use the MAPS program for SEM and STEM, but correlative software is now also available on TEMs, facilitating correlative electron microscopy from the SEM to the TEM level. Additionally, newer microscopes have improved options for low magnification imaging along with specialized software, such as MAPS 3 (TF/FEI Talos 120 keV) and MirrorCLEM (Hitachi), and PictureOverlay (Jeol) software. These technologies will help streamline and possibly even simplify CLEM methods for users moving forward, and will be a future step to advance *i*CLEM methods.

The motivation in designing the *i*CLEM methods presented here has been to offer new tools to identify and understand tissue stem cells under different states, physiological conditions, or to decipher closely related phenotypes. Uncovering the identity of highly plastic cells such as tissue stem cells and tumour cells continues to be an important challenge across a number of organ tissues, in particular, in the mature state. Being able to distinguish stem cell types of interest amongst heterogeneous populations or closely related phenotypes permit researchers to unlock the clues governing in vivo behaviour and function. For example, quantifying the abundance of clusters of progenitor cells using *i*CLEM-SEM between uninjured and injured states, along with population changes in the number of non-cycling radial-glia cells or proliferating radial-glia cells would be valuable to uncover the lineage relationship derived from constitutively quiescent and active radial-glia in the stem cell niche. This paper presents a novel method for studying stem cell compartments at both population and single cell levels that capitalizes on the specificity of fluorescent labelling and EM resolution. These *i*CLEM methods provide a feasible approach readily adaptable to most existing EM microscopes to observe and characterize tissue stem cells within plant and animal models with a combination of large overviews and detailed ultrastructure not previously attainable.

## Materials and methods

### Animals

Experiments were performed using transgenic zebrafish aged 6–10 months, from the Proliferating Cell Nuclear Antigen (PCNA) reporter line Tg(PCNA:GFP). Animals were sacrificed by an overdose of Tricaine (0.4%) diluted in ice-cold facility water and brains processed for *immuno*-Correlative Light and Electron Microscopy (*i*CLEM). All animal experiments were assessed and approved by the Monash University Animal Ethics Committee and were conducted under applicable Australian laws governing the care and use of animals for scientific research.

### Forebrain lesions

Forebrain lesions through the lateral axis of the telencephalon were performed as described previously^22^.

### *Immuno*-correlative light and electron microscopy (*i*CLEM)

#### Tissue processing

##### Tissue Fixation

For all *i*CLEM analyses, the adult zebrafish forebrain of Tg(PCNA:GFP) transgenic fish was dissected out along with the olfactory bulbs for tissue orientation (Fig. 1; step 1), and fixed in 2% PFA, 0.2% Glutaraldehyde (GA) and 4% sucrose in phosphate buffer (PB, 0.1 M, pH 7.4) overnight at 4 °C (Fig. 1; step 2).

##### Tokuyasu embedding & sectioning

Fixed whole forebrains were washed with 0.15% glycine in PBS and infused in 6% gelatin in PB at 37 °C for 60 min followed by infusion of 6% gelatin in PB at 37 °C for 60 min (Fig. 1; step 3). Forebrain gelatin blocks were subsequently cooled to 4 °C for 60 min to allow the gelatin to solidify (Fig. 1; step 4). A post fixation of 0.2% PFA in PB was next performed for 30 min at 4 °C (Fig. 1; step 5). Thereafter, forebrain gelatin blocks were washed thrice with PB and infused with 2.3 M sucrose in PB for 2 days at 4 °C (Fig. 1; step 6). Blocks were mounted on an aluminum pin. Excess sucrose was removed with filter paper and blocks on pins frozen in liquid nitrogen or in the chamber of the cryo ultramicrotome (Fig. 1; step 7).

Frozen blocks were trimmed to the region of interest (ROI) at -100 °C using a Leica UC7/FC7 cryo-ultramicrotome and diamond trimming knife (Diatome; Fig. 1; step 8). Thick sections were collected in a mixture of 2% methylcellulose/2.3 M sucrose (1:1) then placed on an object slide and stained with methylene blue/Azur II solution. After locating the ROI by light microscopy, sections were cut using a diamond immuno knife (Diatome; Fig. 1; step 9). For SEM, semithin 200 nm sections were placed directly in carbon coated, 1µ-slide 2 × 9 well microscope chambers (Ibidi, 81,801; Fig. 1; step 10A). For both STEM and TEM, ultrathin 70 nm sections were cut and placed on 50 mesh copper grids with carbon coated formvar film (Fig. 1; step 10B).

#### Correlative workflow

##### Correlative immuno-labelling for SEM, STEM, and TEM

Correlative immunofluorescent (IF) labelling was carried out as previous^4,10,55^ to detect glial cells and cells actively cycling in sections of the dorsal stem cell niche of the adult zebrafish telencephalon (Fig. 2—SEM; Fig. 4—STEM/TEM). Semithin sections (200 nm; SEM) were IF-labelled directly in Ibidi chambers (Fig. 2; steps 1–2), while ultrathin sections (70 nm; STEM/TEM) were IF-labelled on grids on parafilm (Fig. 1; step 10B; Fig. 4; steps 1–2). Primary antibodies mouse-anti-Glutamine Synthetase (1:500; GS; Merck Millipore, MAB302), biotinylated anti-eGFP (1:300; Rockland, 600–106-215) and rabbit anti-biotin (1:10.000; Rockland, 100–4198) were used to label glial cells and Green Fluorescent Protein (GFP)-labelled proliferating cell nuclear antigen (PCNA), respectively. PCNA labels all cycling cells present in the late G_1_ phase to M-phase of the cell cycle^34,35^. Primary antibodies were detected by goat-anti-mouse AlexaFluor-555 (1:300; ThermoFisher, A21424) and a goat-anti-rabbit AlexaFluor-488 labelled secondary antibody (1:300; ThermoFisher, A11008) followed by 1 µM Hoechst (Invitrogen #33,342) nuclear stain (Fig. 2; step 2; Fig. 4; step 2).

##### Fluorescent optical microscopy for Ibidi chambers (SEM) and grids (STEM/TEM)

Fluorescent imaging of tissue was performed directly in Ibidi chambers in distilled water prior to SEM (Fig. 2; step 3). For STEM and TEM fluorescent microscopy grids were infused in 50% glycerol, placed on a glass bottom 3 cm dish (sections oriented downward) and covered with coverslips (see Fig. 4; step 2). Fluorescent imaging of 200 nm semithin and 70 nm ultrathin forebrain sections of the adult zebrafish telencephalon was performed on a Leica AF6000LX Widefield microscope using a 40 × 0.60 dry lens or a 63 × 1.3 glycerol lens at the Monash Micro Imaging Facility (MMI, Monash University, Clayton Campus, Melbourne). Imaging of complete forebrain sections was accomplished using tile sets and z-stacks at the above magnifications (Fig. 2; step 4; Fig. 4; step 3). Following IF imaging, samples were processed for subsequent EM preparations below.

##### SEM sample preparation using Ibidi chambers

Sample preparation for 200 nm semithin sections for SEM was done in the Ibidi chambers (Fig. 2; step 5–6). Post-fixation steps and rinsing steps in between were performed in sodium cacodylate buffer (pH 7.4) within Ibidi chambers: 2.5% GA overnight at 4 °C, 2% Osmium tetroxide with 1.5% Potassium Ferricyanide for 90 min. at 4 °C, and 1% Tannic acid for 30 min at room temperature. This was completed by incubating sections with 1% Osmium Tetroxide in distilled water for 30 min. at 4 °C (Fig. 2; step 6). Forebrain sections were next punched out of Ibidi chambers with a 3.5 mm diameter biopsy puncher (ProSciTech, T982-35) and stained with Uranyloxalate (pH 7.0) and Methylcellulose/Uranylacetate (pH 4.0) on ice. Finally, sections were looped out, air-dried and mounted on SEM stubs using carbon tabs. Stubs with brain sections were iridium coated at the Monash Centre for Electron Microscopy (MCEM, Monash University, Melbourne), before being mounted on a standard SEM holder (Fig. 2; step 6–7).

##### STEM and TEM sample preparation for grids

Grids were detached from glass bottom dishes by adding distilled water causing the coverslips to float (Fig. 4; step 4). Grids could then be lifted easily. Thereafter, grids were stained with Uranyloxalate (pH 7.0) and Methylcellulose/Uranylacetate (pH 4.0) on ice and looped out and air dried (Fig. 4; step 4). Grids where then placed on a STEM or TEM holder, respectively for imaging (Fig. 4; STEM—step 5A, TEM—step 5B).

#### TEM immuno gold double-labelling workflow

##### Immuno double labelling with gold particles for sequential grids

Immuno double-labeling on grids for TEM was completed as described by Slot and Geuze^13^ (Fig. 6; steps 1–3). In brief, to specifically label radial-glial cells, we used a mouse anti-Glutamine Synthetase (GS) primary antibody (1:500; Millipore, MAB302), followed by a rabbit anti-mouse Ig bridging step (1:1000; Rockland, 610–4120) and protein A Gold 10 nm (1:50; Dept. of Cell Biology, UMC Utrecht, the Netherlands). To prevent non-specific binding of the subsequent protein A Gold particles, 1% GA in PB was used. Next, PCNA of the Tg(PCNA:GFP) transgenic reporter line was labelled with a biotinylated enhanced Green Fluorescent Protein (eGFP) primary antibody (1:500; Rockland, #00–106-215) followed by rabbit anti-Biotin (1:10.000; Rockland, 100–4198), and protein A Gold 20 nm (Dept. of Cell Biology, UMC Utrecht, Netherlands). To stabilize labelling, 1% GA in PB was used. Finally, staining was completed using Uranyloxalate (pH 7.0) and Methylcellulose/Uranylacetate (pH 4.0) on ice, and looped out and air-dried for EM imaging (Fig. 6; steps 3–4).

#### Electron microscopy imaging

SEM and STEM images were taken on a Thermo Fisher/FEI Nova Nano SEM 450, with MAPS 2.0 software used to create tile sets of large areas and correlation. For SEM the retractable BSE detector at 10 keV and 5 mm working distance was used. For STEM a STEM II (HAADF) detector set at 30 keV was used with a working distance of 6.8 mm. TEM images were taken using a Hitachi S-7500 TEM set at 80 keV equipped with a Gatan Multiscan digital camera, as well as using a Jeol JEM-1400 Flash 120 keV TEM. All electron microscopy imaging was completed at the Ramaciotti Centre for Cryo Electron Microscopy (Monash University, Melbourne).

## Supplementary Information

Supplementary Information.

## Data Availability

The datasets generated during and/or analysed during the current study are available from the corresponding authors upon reasonable request.
