# Supplementary material for: TEM, SEM, and STEM-based immuno-CLEM workflows offer complementary advantages
Source: Sci Rep. 2021 Jan 13;11:899. doi: 10.1038/s41598-020-79637-9 (PMC7806999; doi:10.1038/s41598-020-79637-9)
Supplement: Supplementary file 1 — Supplementary Information. [file 41598_2020_79637_MOESM1_ESM.pdf]

Supplementary Information: 1 Table, 2 Figures, 2 Legends

TEM, SEM, and STEM-based immuno-CLEM workflows offer complementary advantages

Authors: Viola Oorschot[1,4#], Benjamin W. Lindsey[2,5#], Jan Kaslin[2*], Georg Ramm[1,3*]

#Co-first authors, *Co-senior authors/corresponding authors

Affiliations:

[1]Ramaciotti Centre for Cryo EM, Monash University, Melbourne, Victoria, 3800, Australia.

[2]Australian Regenerative Medicine Institute, Monash University, Melbourne, Victoria, 3800, Australia.

[3]Department of Biochemistry, Biomedicine Discovery Institute, Monash University, Melbourne, Victoria, 3800, Australia.

[4]Present address: European Molecular Biology Laboratory, Electron Microscopy Core Facility, Heidelberg, Germany.

[5]Present address: Department of Human Anatomy and Cell Science, Rady Faculty of Health Sciences, University of Manitoba, Winnipeg, R3E 0J9, Canada.

Corresponding Authors:

*Georg Ramm: georg.ramm@monash.edu

*Jan Kaslin: jan.kaslin@monash.edu

Supplementary Figure 1. Specificity of immuno-gold labelling of cells in the adult zebrafish forebrain. A-C: Green Fluorescent Protein (GFP)-positive proliferative cell displaying 20 nm gold particles in both the cytoplasm and nucleolus. **D-F:** Glutamine Synthetase (GS)-positive glial cell showing 10 nm gold particles restricted primarily to the cytoplasm of the cell. White boxes depict insets shown at increasing magnification. N, nucleus; ER, endoplasmic reticulum; MVB, multi vesicular body; G, golgi. Scale bars: **A, E,** 1 µm; **B, C, F, G,** 200 nm.

Supplementary Figure 2. Negative labelling control in cells in the adult zebrafish forebrain. A-C: Negative immuno-gold labelling of GFP and GS in mature neurons of the adult brain. White boxes depict insets shown at increasing magnification. Note the slight background labelling for 10 nm gold (green arrowhead) and 20 nm gold (red arrowhead). N, nucleus; M, mitochondrium; G, golgi. Scale bars: **A, E,** 1 µm; **B, C** 200 nm.

Table 1. Benefits and limitations of *i*CLEM using Tokuyasu sample preparation.

Microscopy Technique	Benefits	Limitations
Scanning Electron Microscopy (SEM)	• Thick semithin sections (i.e. 200 nm) produce increased immunofluorescent signal	• Suffers in resolution at higher magnifications (i.e. > 5k) for cellular and organelle detail
	• Absence of grid bars results in no loss of information from tissue sections	
	• Compatible with MAPS software allowing easy correlation between IF and EM images	
	• Large tile sets of region of interest can be examined with potential for quantification	
	• Potential for serial sectioning to yield 3-dimensional reconstructions	
Scanning Transmission Electron Microscopy (STEM)	• Bridges correlative gap between SEM and TEM using MAPS software	• Raster scanning of Tokuyasu sections can more easily damage tissue, keep mag. <8k
	• Provides higher resolution overviews than SEM	• Ultrathin sections (i.e. 70 nm) produce weaker immunofluorescent labelling
	• Permits large tile sets to be scanned equivalent to SEM	• Presence of grid bars occludes tissue regions which may be of interest
Transmission Electron Microscopy (TEM)	• Provides high resolution ultrastructural detail of cellular components and organelles	• Ultrathin sections (i.e. 70 nm) produce weaker immunofluorescent labelling
	• Absence of raster scanning reduces tissue damage observed in STEM	• Presence of grid bars occludes tissue regions which may be of interest
	• Beam illumination of entire field of view maintains tissue integrity during imaging	• No correlative software currently is available at the TEM level

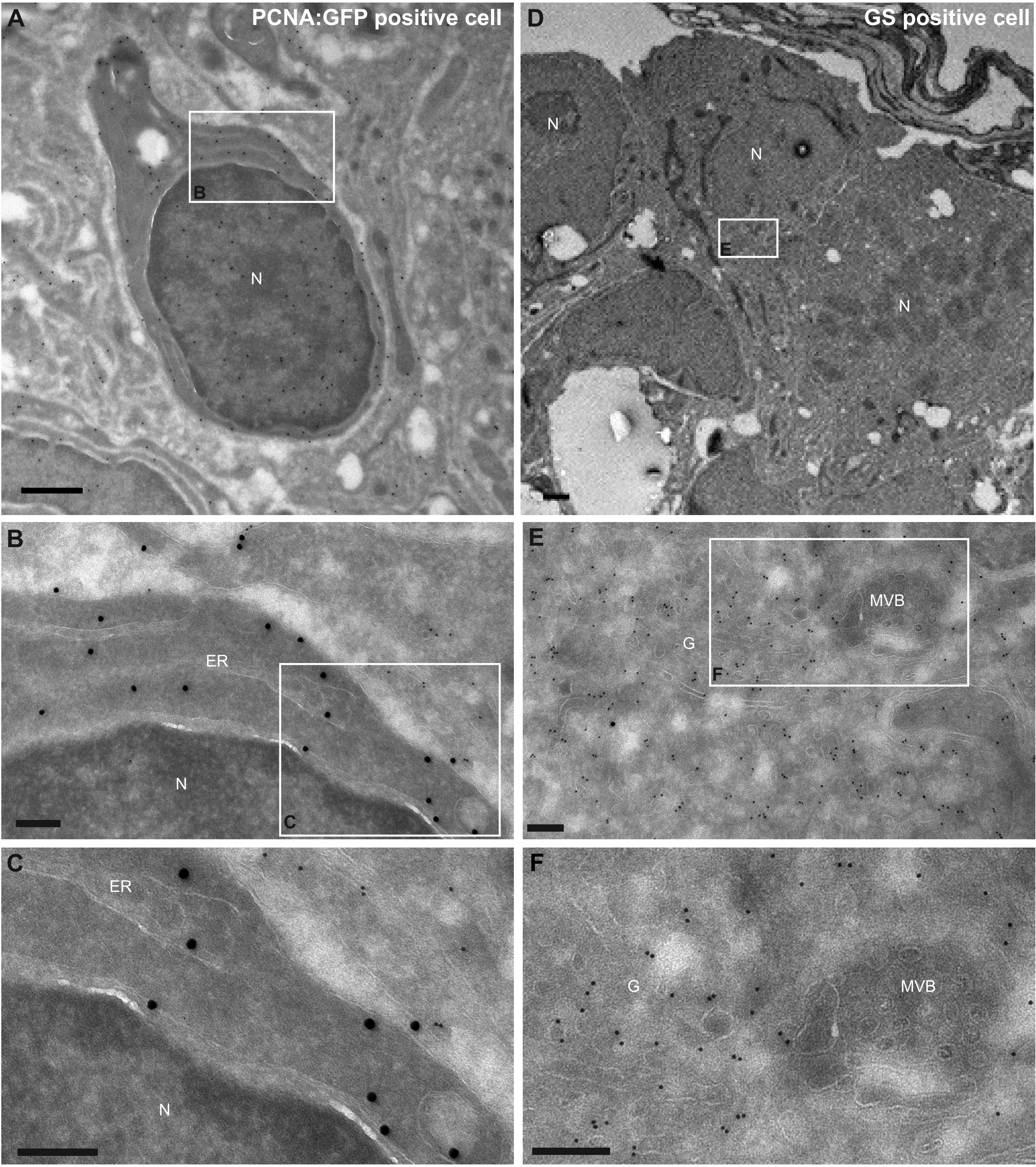

Suppl. Figure 1

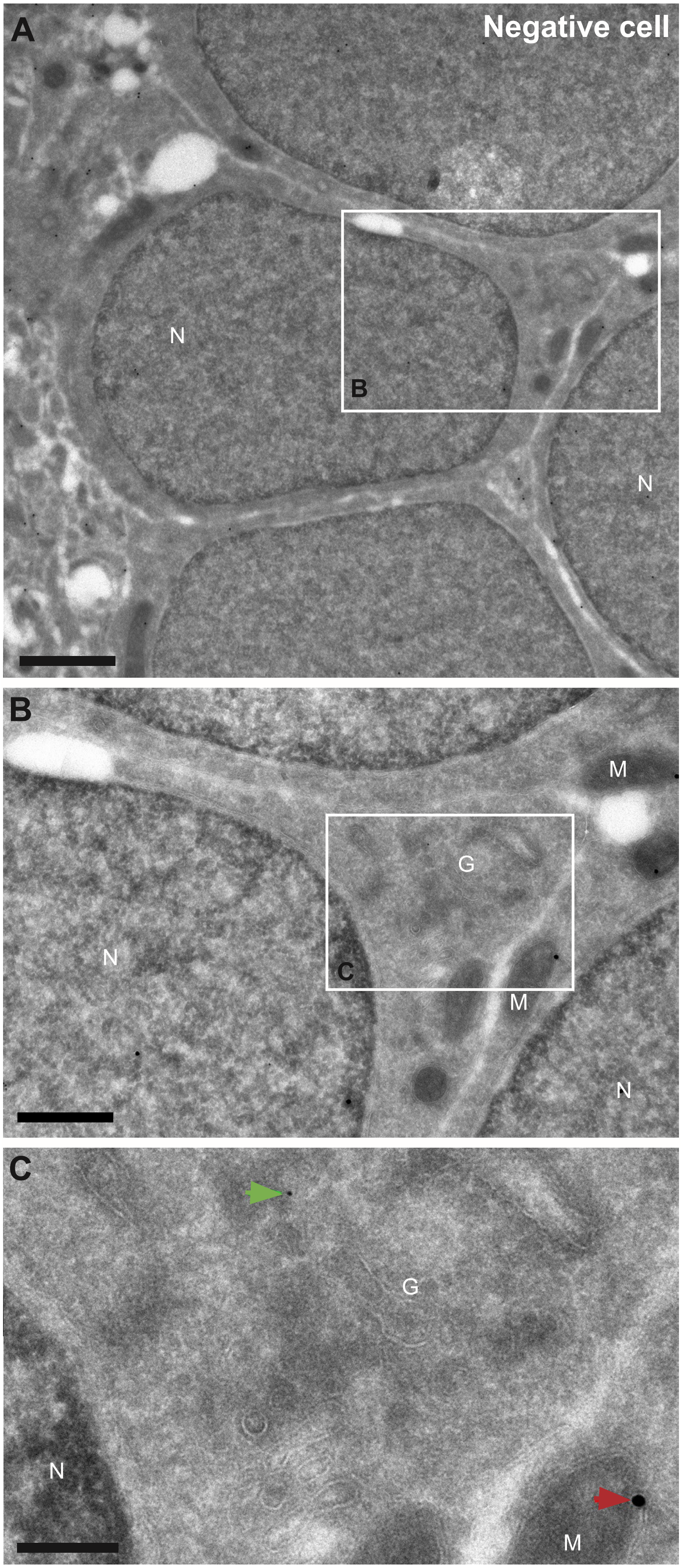

Suppl. Figure 2
